# Opioid prescriptions for individuals receiving workers’ compensation in Michigan

**DOI:** 10.1371/journal.pone.0272385

**Published:** 2022-08-09

**Authors:** Kenneth D. Rosenman, Ling Wang

**Affiliations:** Department of Medicine, Michigan State University, East Lansing, Michigan, United States of America; Children’s Hospital Srebrnjak: Djecja Bolnica Srebrnjak, CROATIA

## Abstract

**Purpose:**

We evaluated the prevalence of opioid prescriptions after injury and associated characteristics among workers receiving workers’ compensation for a lost work time injury.

**Methods:**

Injured workers identified in Michigan’s Workers’ Compensation records from 2016 to 2018 were linked to the opioid prescription history in the Michigan Automated Prescription System.

**Results:**

Among the 46,934 injured workers with paid claims, the prevalence of receiving an opioid prescription, morphine milligram equivalents (MME) per prescription, number of opioid prescription and probability of receiving opioids prescription>90 days after injury decreased from 2016–2018. Despite the decrease over 50% of the injured workers received an opioid prescription. Being over 34 years, a male, having had an opioid prescription before the injury, working in construction or having an amputation or sprain/strain of the shoulder had a significantly higher probability of receiving an opioid prescription, a higher MME per prescription, a higher number of opioid prescriptions and a higher probability having opioids prescription >90 days after the injury.

**Conclusions:**

Even though opioid prescribing patterns generally decreased from 2016 to 2018 (64.5–52.8%), injured workers in Michigan had a higher prevalence of opioid prescription after injury, than those reported from other states.

## Introduction

In 2016, in response to concerns about the increase in the occurrence of addiction to opioids and opioid -related deaths, CDC issued guidelines about the use of opioids for chronic non-cancer related pain [1]. Two years earlier, the organization representing occupational medicine physicians had issued guidelines for the use of opioids for work-related injuries [2]. In the United States, prescription rates for opioids peaked in 2012 and decreased 44% by 2020 [3]. A similar trend was seen in Michigan where the opioid dispensing rate decreased from 100.7 per 100 persons in 2012 to 54.4 per 100 persons in 2020 [3]. In 2020, Michigan had the 12^th^ highest opioid dispensing rate in the United States [4] and in 2019, with 2,385 deaths, the 21^st^ highest rate of drug overdose deaths [5]. In 2019, the number of opioid prescription related overdose deaths in Michigan was 454, which was decreased from 678 in 2016 [6]. However, in 2020, opioid overdose deaths in Michigan increased 16.2% [7].

In 2018, Michigan began to require a provider writing an opioid prescription to obtain a signature on the Start Talking Consent Form; obtain and review a Michigan Automated Prescription System (MAPS) report for any patient before prescribing a controlled substance for a quantity greater than three days; provide follow-up care or referral to another provider to monitor the efficacy of the controlled substance in treating the patient’s condition; prescribe no more than a seven day supply of an opioid to patients being treated for acute pain; and discuss the dangers of opioid addiction, how to dispose of an expired, unused controlled substance, and the Michigan laws involving delivery of a controlled substance, as well as the short term and long-term effects of exposing a fetus to an opioid [8].

In June 2015, Michigan initiated specific regulations related to workers’ compensation reimbursement for opioids that continued to be prescribed for more than 90 days after a work-related injury [9]. These regulations required a written report every 90 days that included: (a) A review and analysis of the relevant prior medical history and MAPS; (b) A summary of conservative care rendered to the worker that focused on increased function and return to work; (c) A statement on why prior or alternative conservative measures were ineffective or contraindicated; (d) A statement that the attending physician has considered the results obtained from appropriate industry accepted screening tools to detect factors that may significantly increase the risk of abuse; (e) A treatment plan every 6 months that included: (i) Overall treatment goals and functional progress; (ii) Periodic urine drug screens; (iii) An effort to reduce pain through the use of non-opioid medications, alternative non-pharmaceutical strategies, or both; (iv) Consideration of weaning the injured worker from opioid use; and (f) Every six months an opioid treatment agreement signed by the worker/patient and the doctor [9].

Even before this new regulation had time to be effective, a report from the Workers’ Compensation Research Institute (WCRI) on opioid prescriptions per workers’ compensation claims from 2010/2012 to 2013/2015 showed that Michigan had a 37% decrease in the average morphine milligram equivalents (MME) per claim, which was the largest decrease in the 26 states studied [10]. Since then, it is unknown how prescribing practices have changed for work-related injuries in Michigan. Additionally, there has been no previous analysis of all opioids prescribed to injured workers, which would include those not paid for by workers’ compensation. This manuscript examines all opioid prescriptions to workers receiving workers’ compensation in Michigan.

## Methods

### Data sources

The Michigan Workers’ Disability Compensation Act (WDCA) covers all Michigan workers except: Federal employees; railroad employees; seamen on navigable waters; workers who load and unload water vessels; private employers who employ less than three workers if none of the worker worked more than 35 hours per week for 13 weeks or longer in the preceding 52 weeks; and the self-employed.

Both self-insured employers and insurance companies are required to report immediately, to the agency, on form WC-100, or its electronic equivalent, all injuries, including diseases, which arise out of and in the course of the employment, or on which a claim is made, and result in any of the following: (a) Disability extending beyond 7 consecutive days, not including the date of injury. (b) Death. (c) Specific losses. Only workers’ compensation claims that receive wage replacement are computerized by WDCA and therefore only these wage replacement claims could be accessed for analysis. To receive wage replacement, a worker must be off work seven days or more in a row. The seven days in a row can be five workdays and normal time off, such as a two-day weekend.

The Michigan Automated Prescription System (MAPS) has data on all dispensed and prescribed controlled substances (DEA schedule 2–5 drugs). Board of Pharmacy Administrative Rule 338.3162b requires all pharmacies, practitioners, and veterinarians who dispense schedules 2–5 controlled substances to electronically report daily prescription data to MAPS. Exemptions to these requirements include controlled substances administered to patients, samples of controlled substances provided to a patient, and controlled substances that are dispensed by a physician at a medical facility for a maximum of 48 hours.

### Data matching

The 2016, 2017, and 2018 WDCA data on paid claims were matched to the MAPS data from 1/1/2003 to 9/2020. A contractor of MAPS received the WDCA data with personal identifiers, performed the data linkage steps with MAPS data, and then removed all patient-level identifiers from the linked dataset prior to transferring the data back to MSU. Data were first matched deterministically on social security number and then by first name, last name, and date of birth. Workers’ compensation paid claims that could not be deterministically matched to MAPS records underwent a probabilistic matching process using first name, last name, day, month, and year of birth, gender, zip code, and the last four digits of the social security number. Variables used for matching were standardized prior to analysis, such as removing non-alphanumeric characters from names, removing leading or trailing whitespace, and excluding values used to identify missing or incomplete data (e.g. 999999999 for a social security number).

### Statistical analysis

The outcomes evaluated were the percent of workers who received an opioid prescription, the number of opioid prescriptions per claim, the number of claims with an opioid prescription >90 days’ duration, and the mean and median MME per prescription. These outcomes were evaluated for the overall data, by the year of injury (2016, 2017 and 2018); by three age categories (15–34, 35–54 and ≥55); by gender (male/female); by receipt of an opioid prescription prior to the injury (yes/no); by residence status (rural, urban or metro); by type of injury; and by industry. If an injury occurred in the second half of the calendar year, the percent receiving an opioid prescription at one week, one month, and six months would be counted in the year of injury even if the opioid prescription was in the next year. Differences were examined by chi-square tests of association for categorical data, chi-square tests of trend for discrete data, and by t-tests for continuous data.

Residence in a rural or urban area was based on the county that contained the entire or most of the health care provider’s zip code. If this was not available than the worker’s residence was used. The 2013 National Center for Health Statistics Urban-Rural Classification Scheme for Counties was used to classify each county as an urban or rural area.

To reduce autocorrelation within the data, only the first injury was included in the regression analysis among workers with more than one injury during the surveillance period (Tables 2 and 4). Among workers who received at least one opioid prescription, similar regression models were used to test for factors associated with higher doses (MME) and increased number of opioid prescriptions. Since the data were highly right skewed, quantile regression was used to model the risk factors associated with median of morphine milligram equivalents per prescription. Zero-truncated Poisson regression was used to model the number of prescriptions within six months of injury since this was count data and we only used the workers who received at least one opioid prescription after their injury.

Kendall Tau correlation was used to determine if the proportion of work-related injury claims, which received an opioid prescription, changed over time. Kendall Tau correlation was also used to determine if the mean daily MME changed over time among workers with one or more opioid prescription within six months of a work-related injury.

The analyses presented in Tables 1, 3 and 5 were based on claims while the analysis in Tables 2 and 4 were based on individual workers. For the analyses performed by individual workers, we performed a sensitivity analysis to determine if the results changed if we excluded the 1,428 (3.06%) of workers who had more than one injury from 2016–2018.

**Table 1:** Description of morphine milligram equivalents (MME), number of opioid prescriptions per claim and number of claims with opioid prescriptions >90 days duration within 6-months of an injury among workers, who received a paid wage replacement WC claim, by year from 2016–2018 (n = 28,607 claims).

Variable	Year of Injury	Mean (SD)	Median	Maximum
MME per prescription^1^ (Milligram)	2016	8.7(35.1)	5.7	2,495.8
	2017	9.2(36.3)	5.6	1,974.3
	2018	8.5(21.3)	5.4	581.1
	2016–2018	8.8(31.9)	5.6	2,495.8
Number of opioid prescriptions per claim^2^	2016	3.1(3.0)	2	34
	2017	3.0(2.9)	2	44
	2018	2.7(2.7)	2	33
	2016–2018	3.0(2.9)	2	44
Number of claims with opioid prescriptions >90 days duration^3^	**Year of Injury**	**N**	**%**	
	2016	3,074	29.8	
	2017	2,518	27.3	
	2018	2,012	22.2	
	2016–2018	7,604	26.6	

^1^ Kendall Tau coefficient for mean from 2016 to 2018 is -0.018, P = 0.001.

^2^ Kendall Tau coefficient for mean from 2016 to 2018 is -0.060, P<0.001.

^3^ Kendall Tau Coefficient percent from 2016 to 2018 is -0.067, P<0.001.

**Table 2:** Percent of workers with an opioid prescription, median MME per prescription, median number of opioid prescriptions per worker and percent of workers with opioid prescriptions >90 days duration by age, gender, previously prescribed opioids, and urban/rural residence of workers within 6-months of an injury with a paid wage replacement WC claim, 2016–2018 combined (n = 46,714 workers).

	No opioid prescription filled within 6 months (n = 18,969 workers)	Had opioid prescription filled within 6 months (n = 27,745 workers)	% workers with an opioid prescription	Had opioid prescription filled within 6 months of Injury (n = 27,745 workers)		
				MME per prescription (Milligram)	Number of opioid prescriptions per worker	Percent of workers with opioid prescriptions >90 days duration
**Age (years)** ^**1**^	**N**	**N**		**Median**	**Median**	**%**
15–34	5,980	6,285	51.2	5	1	18.8
35–54	8,364	13,003	60.9	5.7	2	29.0
≥55	4,625	8,457	64.7	5.7	2	28.3
**Gender** ^**2**^						
Female	7,813	9,586	55.1	5.2	2	30.2
Male	11,291	18,163	61.7	5.8	2	24.6
**Received Opioid prescriptions before injury** ^**3**^						
Yes	9,535	18,317	65.8	5.7	2	33.1
No	9,434	9,428	50.0	5	1	13.6
**Residence status** ^**4**^						
Rural Area	1,041	1,403	57.4	5.5	2	29.7
Urban Area	3,094	4,926	61.4	5.5	2	24.0
Metro Area	14,834	21,416	59.1	5.6	2	26.9

^1^ Different proportion of opioid prescriptions (P<0.001); different median MME (P<0.001); different median number of opioids prescriptions (P<0.001); different proportion of opioid prescriptions duration>90 days (P<0.001).

^2^ Different proportion of opioid prescriptions (P<0.001); different median MME (P<0.001); different median number of opioids prescriptions (P<0.001); different proportion of opioid prescriptions duration>90 days (P<0.001).

^3^ Different proportion of opioid prescriptions (P<0.001); different median MME (P<0.001); different median number of opioids prescriptions (P<0.001); different proportion of opioid prescriptions duration>90 days (P<0.001).

^4^ Different proportion of opioid prescriptions (P<0.001); different median MME (P = 0.002); different median number of opioid prescriptions (P = 0.01); different proportion of opioid prescriptions duration>90 days (P<0.00).

## Results

**Fig 1:**
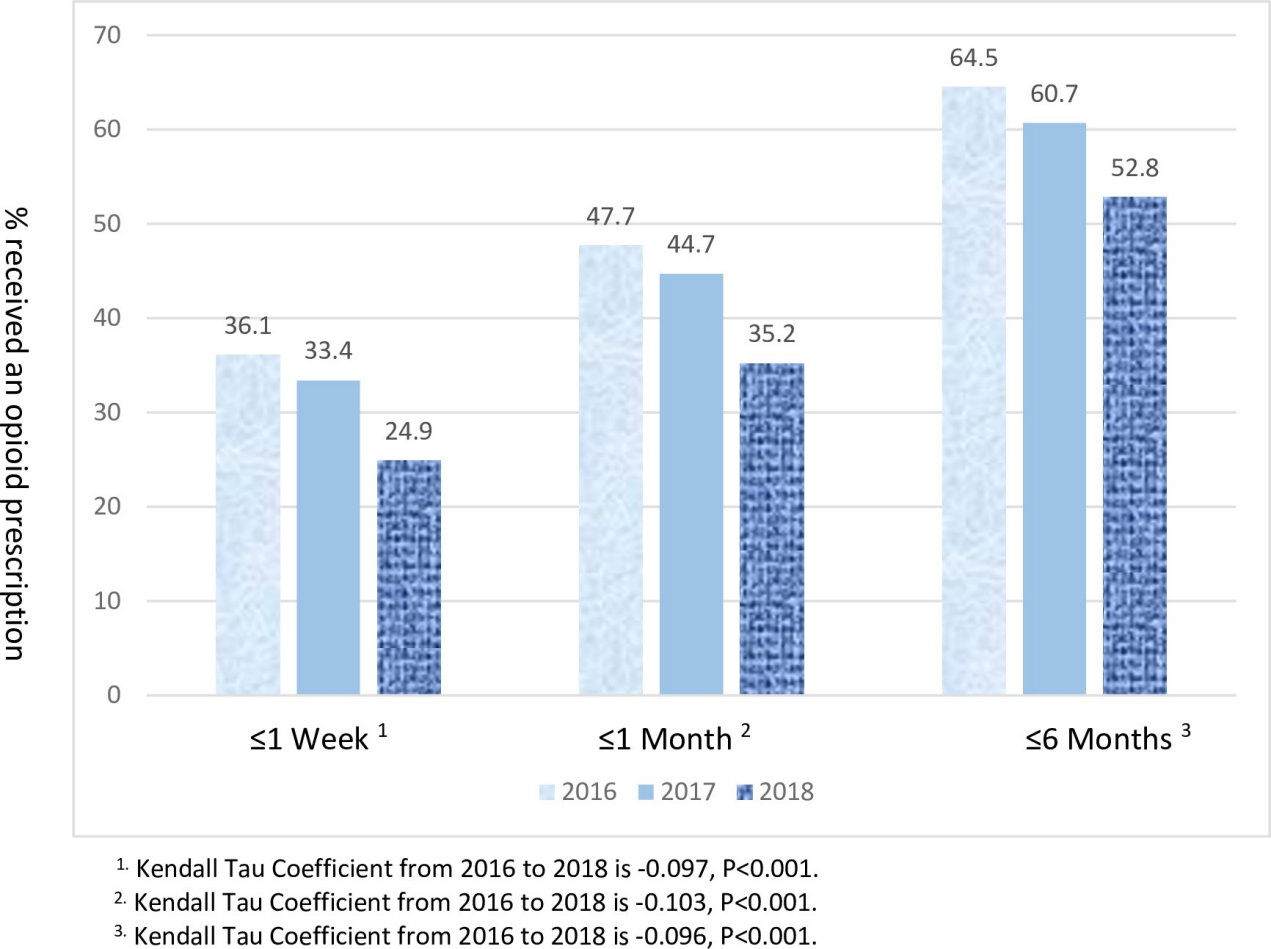
Percent of workers who received an opioid prescription within I week, I month and 6 months of an injury for which they received a paid wage replacement WC claim, by year and combined 2016–2018 (n = 46,934). ^1.^ Kendall Tau Coefficient from 2016 to 2018 is -0.097, P<0.001. ^2.^ Kendall Tau Coefficient from 2016 to 2018 is -0.103, P<0.001. ^3^ Kendall Tau Coefficient from 2016 to 2018 is -0.096, P<0.001.

The number of injured workers in 2016, 2017 and 2018 with a paid WC claim and the number and percent with an opioid prescription within one week, one month and six months after the injury are shown in Fig 1. There was a statistically significant downward trend (p<0.001) in the percentage of injured workers who received an opioid prescription over the three years for all three time periods after their injury.

The remainder of the analyses were conducted using an opioid prescription within six months of the injury.

Table 1 shows there was a statistically significant decrease in the mean MME per prescription, the mean number of opioid prescriptions per claim and the percent of claims with opioid prescriptions > 90 days’ duration from 2016–2018 (Kendall Tau coefficients are -0.02, -0.06 and -0.07 respectively with P-values≤0.001).

Table 2 shows that the percent of workers with an opioid prescription, differed statistically by age, gender, received an opioid prescription prior to the work-related injury, and residence. This was also found for MME per prescription, number of opioid prescriptions per workers and number of workers with opioid prescriptions >90 days’ duration.

Table 3 shows that the percent of claims with an opioid prescription, differed statistically by both injury and industry (p<0.001). Among claims with received an opioid prescription, the median MME, number of opioid prescriptions per claim and the number of claims with an opioid prescription >90 days differed statistically by both injury and industry (p<0.001).

**Table 3:** Percent of claims with an opioid prescription, median MME per prescription, median number of opioid prescriptions per claim and percent of claims with opioid prescriptions >90 days duration by injury type and industry within 6 months of a paid wage replacement WC claim, 2016–2018 combined (n = 48,453 claims).

Injury^1^	No opioid prescription filled within 6 months (n = 19,846 claims)	Had opioid prescription filled within 6 months (n = 28,607 claims)	% of claims with an opioid prescription	Opioid prescription filled within 6 months of Injury (n = 28,607 claims)		
				MME per prescription (Milligram)	Number of opioid prescriptions per claim	Percent of claims with opioid prescriptions >90 days duration
				Median	Median	%
Amputations	75	369	83.1	5.8	2	18.2
Abrasions/Cuts/Lacerations/Bites	1,206	1,963	61.9	5.0	2	17.3
Crush/Contusions	2,472	2,953	54.4	5.5	2	29.9
Fracture/Dislocations	2,539	6,571	72.1	5.6	2	24.2
Sprain/Strain/Hernia/Inflam	3,504	3,567	50.4	5.4	2	27.0
Sprains and Strains—Back	2,665	2,491	48.3	5.2	2	33.0
Sprains and Strains—Shoulder	1,502	2,700	64.3	6.3	2	31.9
Sprains and Strains—Knee	1,377	2,258	62.1	5.5	2	22.6
Sprains and Strains—Arm/Hand	1,528	1,947	56.0	5.6	2	26.3
Burns-Chemical/Heat/Electrical	291	451	60.8	5.6	2	16.2
Concussions	382	239	38.5	5.0	2	24.7
Diseases	162	75	31.6	5.0	1	21.3
Misc. ill-defined injuries	2,143	3,023	58.5	5.6	2	30.0
**Industry** ^**2**^						
Agriculture/Forestry/Fishing	191	363	65.5	5.0	2	22.0
Construction	1,273	2,598	67.1	6.1	2	26.1
Public Safety	477	458	49.0	5.6	2	27.3
Healthcare & Social Assistance	2,819	3,370	54.5	5.3	2	31.1
Manufacturing	3,192	6,095	65.6	5.6	2	25.3
Oil & Gas Extraction	13	16	55.2	6.0	2.5	25.0
Mining (except Oil/Gas Services)	12	30	71.4	6.3	2	16.7
Services (except Public Safety)	7,076	9,482	57.3	5.6	2	26.4
Transportation/Warehousing/ Utilities	1,931	2,045	51.4	5.7	2	27.1
Wholesale & Retail Trade	2,842	4,129	59.2	5.5	2	25.4

^1^ Significantly different proportion of opioid prescriptions (P<0.001); significantly different medians of MME (P<0.001); significantly different medians of number of opioid prescriptions (P<0.001); significantly different proportion of opioid prescriptions duration>90 days (P<0.001).

^2^ Significantly different proportion of opioid prescription (P<0.001); significantly different medians of MME (P = 0.02); significantly different medians of number of opioid prescription (P<0.001); significantly different proportion of opioid prescriptions duration>90 days (P<0.001).

Table 4 shows that controlling for all characteristics, the predicted probability of an injured worker receiving an opioid prescription, predicted median MME, predicted number of opioid prescriptions per worker and the predicted probability of opioid prescriptions >90 days. It is found that working in construction industry or injury type as Sprains and Strains- Shoulder leading to significantly higher probability of receiving opioids prescription after injury, higher MME per prescription, higher number of opioids prescription and higher probability having opioids prescription >90 days after injury compared to other industries consistently.

**Table 4:** Predicted probability of an injured worker receiving an opioid prescription, predicted median MME per prescription, predicted number of opioid prescriptions per injured worker and predicted probability of an opioid prescriptions >90 days duration within 6 months of a work-related injury based on multivariate regression.

Characteristics		Predicted probability of receiving an opioid prescription (95% C. I.)^2^	Predicted median MME per prescription (95% C. I.)^3^	Predicted number of opioid prescriptions per injured worker (95% C. I.)^4^	Predicted probability of opioid prescriptions >90 days duration (95% C. I.)^5^
**Year**	2016	▲0.66(0.65–0.66)	▲5.73(5.68–5.78)	▲3.03(2.99–3.06)	▲0.31(0.3–0.32)
	2017	▲0.61(0.60–0.61)	5.60(5.55–5.65)	2.79(2.75–2.83)	0.27(0.26–0.28)
	2018	▼0.52(0.51–0.53)	▼5.47(5.42–5.53)	▼2.36(2.33–2.40)	▼0.21(0.2–0.22)
**Gender**	Female	▼0.56(0.56–0.57)	▼5.37(5.31–5.42)	2.68(2.64–2.72)	▲0.28(0.27–0.29)
	Male	▲0.61(0.61–0.62)	▲5.74(5.70–5.78)	2.77(2.75–2.8)	0.26(0.25–0.26)
**Age**	15–34	▼0.53(0.52–0.53)	▼5.42(5.35–5.48)	2.55(2.47–2.64)	▼0.20(0.19–0.21)
	35–54	▲0.61(0.60–0.62)	5.67(5.63–5.72)	▲2.91(2.88–2.94)	▲0.28(0.28–0.29)
	> = 55	▲0.64(0.63–0.64)	5.66(5.60–5.71)	▼2.61(2.55–2.67)	0.28(0.27–0.29)
**Opioids prescription before injury**	No	▼0.49(0.48–0.50)	▼5.41(5.36–5.46)	▼1.80(1.77–1.84)	▼0.14(0.13–0.15)
	Yes	▲0.67(0.66–0.67)	▲5.71(5.68–5.75)	▲3.23(3.20–3.26)	▲0.33(0.32–0.34)
**Residence Rural/Urban Status**	Metro	0.59(0.59–0.60)	5.63(5.60–5.67)	2.74(2.72–2.76)	0.27(0.26–0.27)
	Rural	0.58(0.56–0.60)	5.57(5.43–5.70)	▲2.95(2.85–3.04)	▲0.29(0.27–0.32)
	Urban	0.61(0.6–0.62)	5.53(5.45–5.60)	2.67(2.62–2.72)	▼0.24(0.23–0.25)
**Industries**	Agriculture Forestry & Fishing	▲0.65(0.61–0.69)	5.53(5.25–5.80)	2.75(2.56–2.95)	0.26(0.21–0.30)
	Construction	▲0.65(0.63–0.67)	▲5.98(5.88–6.09)	▲3.11(3.04–3.19)	▲0.29(0.28–0.31)
	Public Safety	▼0.54(0.51–0.57)	5.64(5.39–5.89)	2.68(2.51–2.84)	0.26(0.22–0.30)
	Healthcare & Social Assistance	▼0.58(0.56–0.59)	5.57(5.47–5.66)	2.76(2.69–2.82)	0.27(0.26–0.29)
	Manufacturing	▲0.64(0.63–0.65)	5.54(5.47–5.61)	2.69(2.64–2.73)	0.26(0.25–0.27)
	Oil & Gas Extraction	0.50(0.33–0.68)	5.77(4.49–7.06)	3.42(2.46–4.38)	0.30(0.07–0.52)
	Mining	0.68(0.54–0.82)	5.91(4.97–6.85)	2.74(2.14–3.35)	0.16(0.04–0.29)
	(except Oil & Gas Services)				
	Services (except Public Safety)	▼0.58(0.57–0.59)	5.61(5.55–5.66)	▼2.66(2.63–2.70)	0.26(0.25–0.27)
	Transportation	▼0.52(0.51–0.54)	5.55(5.43–5.66)	2.76(2.68–2.84)	0.26(0.25–0.28)
	/Warehousing/Utilities				
	Wholesale & Retail Trade	0.60(0.59–0.61)	5.54(5.46–5.62)	2.75(2.69–2.80)	0.26(0.25–0.28)
**Injury Type**	Amputations	▲0.83(0.80–0.87)	▲5.99(5.72–6.26)	▲2.97(2.77–3.17)	0.22(0.18–0.27)
	Abrasions/Cuts/Lacerations/Bites	▲0.63(0.62–0.65)	▼5.32(5.20–5.44)	▼2.36(2.28–2.44)	▼0.2(0.18–0.22)
	Crush/Contusions	▼0.55(0.54–0.57)	5.53(5.43–5.62)	▲2.92(2.86–2.99)	▲0.29(0.28–0.31)
	Fracture/Dislocations	▲0.72(0.71–0.73)	5.61(5.55–5.68)	▲2.97(2.93–3.02)	0.25(0.24–0.26)
	Sprain/Strain/Hernia/Inflam.	▼0.51(0.50–0.53)	5.50(5.41–5.59)	▼2.50(2.45–2.56)	0.26(0.24–0.27)
	Sprains and Strains—Back	▼0.49(0.48–0.51)	▼5.42(5.31–5.52)	▲2.94(2.86–3.01)	▲0.31(0.29–0.33)
	Sprains and Strains—Shoulder	▲0.64(0.62–0.65)	▲6.27(6.17–6.37)	▲2.89(2.82–2.96)	▲0.31(0.29–0.33)
	Sprains and Strains—Knee	▲0.62(0.60–0.63)	5.55(5.44–5.66)	▼2.26(2.19–2.33)	▼0.22(0.2–0.24)
	Sprains and Strains—Arm/Hand	▼0.56(0.54–0.57)	5.64(5.52–5.76)	▼2.37(2.30–2.45)	0.25(0.23–0.27)
	Burns-Chemical/Heat/Electrical	0.63(0.59–0.66)	5.62(5.38–5.86)	▼2.44(2.28–2.61)	▼0.19(0.15–0.23)
	Concussions	▼0.40(0.36–0.44)	5.36(5.03–5.70)	▼2.31(2.11–2.52)	0.23(0.18–0.28)
	Diseases	▼0.32(0.27–0.38)	5.28(4.67–5.88)	▼2.25(1.87–2.62)	0.21(0.12–0.29)
	Misc ill-defined injuries	0.59(0.57–0.60)	5.58(5.48–5.67)	▲2.94(2.88–3.01)	▲0.29(0.28–0.31)
**Overall**		0.59(0.59–0.60)	5.61(5.58–5.64)	2.74(2.72–2.76)	0.26(0.26–0.27)

^1.^ Non-overlapping 95% CIs are statistically differences at a significance level α = 0.05 with ▲indicates significantly higher than overall values and ▼indicates significantly lower than overall values.

^2.^ Predicted results are based on logistic regression with sample size = 46,714 injured workers.

^3.^ Predicted results are based on quantile regression with sample size = 27,745 injured workers.

^4.^ Predicted results are based on zero truncated Poisson regression with sample size = 27,745 injured workers.

^5.^ Predicted results are based on logistic regression with sample size = 27,745 injured workers.

Table 5 shows the percent of claims with an opioid prescription and the mean number of opioids prescriptions by injury type over the three years. The decrease was not significantly different by injury except for amputations which had less of a decrease in the percent claims with an opioid prescription than other injuries or overall.

**Table 5:** Percent of claims with an opioid prescription and number of opioids prescriptions within 6 months of a paid wage replacement WC claim by injury type and year, 2016–2018.

Injury Type	% of claims with an opioid prescription			Predicted annual change of % opioid prescription 2016–2018 (95% C.I.)^1^	Number of opioids prescriptions (mean)			Predicted annual change in number of prescriptions 2016–2018 (95% C.I.)^1^
	2016	2017	2018		2016	2017	2018	
**Amputations**	87.4	85.0	77.1	▼-3.5% (-4.2%,-2.9%)	2.9	3.0	2.8	-0.26(-0.29,-0.23)
**Abrasions/Cuts/Lacerations/Bites**	65.8	63.7	56.4	-5.9% (-6.5%,-5.4%)	2.8	2.4	2.3	-0.21(-0.24,-0.19)
**Crush/Contusions**	59.2	57.8	47.5	-6.3% (-6.8%,-5.7%)	3.2	3.2	3.0	-0.28(-0.31,-0.25)
**Fracture/Dislocations**	76.2	73.1	67.4	-5.0% (-5.5%,-4.6%)	3.4	3.1	2.8	-0.27(-0.30,-0.25)
**Sprain/Strain/Hernia/Inflam of Nerves—All**	55.5	52.4	44.3	-6.3% (-6.9%,-5.8%)	2.9	2.8	2.7	-0.24(-0.27,-0.22)
**Sprains and Strains—Back**	55.0	52.2	38.9	-6.3% (-6.9%,-5.8%)	3.4	3.4	2.8	-0.29(-0.32,-0.26)
**Sprains and Strains—Shoulder**	69.7	65.8	58.5	-5.8% (-6.3%,-5.3%)	3.5	3.3	2.7	-0.28(-0.31,-0.25)
**Sprains and Strains—Knee**	67.3	62.3	57.1	-5.9% (-6.5%,-5.4%)	2.8	2.6	2.3	-0.22(-0.24,-0.20)
**Sprains and Strains—Arm/Hand**	62.2	57.2	49.8	-6.2% (-6.8%,-5.7%)	2.8	2.8	2.4	-0.23(-0.26,-0.21)
**Burns-Chemical/Heat/Electrical**	67.8	65.3	50.4	-6.0% (-6.6%,-5.4%)	2.6	2.8	2.3	-0.22(-0.24,-0.19)
**Concussions**	47.6	40.2	28.5	-6.0% (-6.6%,-5.5%)	2.9	2.4	2.5	-0.23(-0.26,-0.20)
**Diseases**	36.2	28.6	28.8	-5.5% (-6.2%,-4.7%)	2.6	2.6	2.5	-0.22(-0.26,-0.18)
**Misc. ill-defined injuries**	66.2	57.5	51.3	-6.1% (-6.7%,-5.6%)	3.2	3.4	2.9	-0.28(-0.31,-0.25)
**Overall**	64.5	60.7	52.8	-5.9% (-6.4%,-5.4%)	3.1	3.0	2.7	-0.26(-0.29,-0.23)

^1.^ Predicted values are obtained based on logistic regressions on year and injury type.

^2.^ Predicted values are obtained based on zero truncated Poisson regressions on year and injury type.

Tables 2 and 4, were reanalyzed to exclude the 1,428 workers with more than one work-related injury. These analyses (not shown) did not differ from the results with these 1,428 workers included.

## Discussion

Over half (52.8%) of workers in Michigan with paid wage replacement claims received opioids in 2018, down from 64.5% in 2016 and 60.7% in 2017 This contrasts with 2006–2010 data from the largest workers’ compensation insurance company in Michigan that reported that 27% of wage replacement claims received an opioid prescription [11]. Prescription rate data paid for by WC among WC recipients has been reported in other states; 19.2% in Ohio (2008–2009), 42% in Washington (2002–2005) and 46.4% in Louisiana (1999–2002) [12–14]. Opioid prescription rates were higher during these earlier time periods but we suspect that opioid prescription rates only paid for by WC underrepresent the true opioid prescription rate to injured workers, which would have been even greater in these earlier time periods if opioid prescriptions written by all health care providers to the injured worker could have been assessed. We are only aware of one other state, Tennessee, where the state-wide prescription monitoring data for all providers were matched with WC data [15]. The percentage of individuals on WC in Tennessee for the years 2013–2015 who received an opioid prescription was 21.7–23.4% at one week, 28.4–30.7% at one month and 31.8–34.3% at six months. The percentage of injured workers in Tennessee receiving opioid prescriptions was lower than in Michigan, which maybe because the WC claim data used in Tennessee included less severe injuries since the Tennessee results were for all reported work-related injuries regardless of claim status and whether or not they had lost work time. The opioid prescription rate in Michigan for work-related injuries were only among those with the most severe injuries who were off work for seven or more days. This presumably explains the higher percentage of injured workers receiving opioids in Michigan even though the overall opioid prescription rate in Tennessee is 27% greater than in Michigan [4].

From 2016–2018, there was a statistically significant decrease in the number of prescriptions for opioids, the mean number of opioid prescriptions per claim and the percent of claims with opioid prescriptions > 90 days’ duration. The regulations instituted for prescribing opioids to all patients and those specifically for patients receiving workers’ compensation presumably contributed to these decreases [8, 9].

Multiple factors were associated with a statically increased opioid prescription rate, increased MME per prescription, and number of opioid prescriptions per worker: older more than young; men more than women; previously received an opioid prescription; living in an urban area (Table 2), injury type (amputations and fractures/dislocations); and industry (Mining, Construction, Manufacturing and Agriculture) (Table 3). The differences in the number of claims with opioid prescriptions >90 days’ duration generally were similar to the other outcomes except men, living in an urban area, amputations, and mining had a lower percent with opioid prescriptions >90 days’ duration (Table 3). To examine the effect of these factors, we used multivariate logistic regression to predict the probability of an opioid prescription (Table 4). Age, gender and previously received an opioid continued to be a factor, additional injuries were identified (Abrasions/Cuts/Lacerations/Bites, Sprains and Strains–Shoulder and Sprains and Strains–Knee) while mining was not significant. Public Safety, Healthcare and Social Assistance, Services, and Transportation/Warehousing/Utilities had significantly lower prescription rates. Crush/Contusions, Sprains and Strains–Back, Sprains and Strains—Arm/Hand, Concussions, and Diseases, had significantly lower prescription rates (Table 4)

Most of the previous publications on opioid prescription and work-related injuries have used information present in workers’ compensation claims data and accordingly have missed opioid prescriptions that were not paid for by workers’ compensation. The higher opioid prescription rate (52.8%) we found in 2018 for injured Michigan workers in comparison to lower percentages from earlier years in Michigan (27%, 2006–2010) and in other states (19.2–46.4%,1999–2009) which were based on workers’ compensation data suggest that workers are receiving opioids from providers outside of the workers’ compensation system. Given the overall downward trend in opioid prescriptions for all patients, one would have expected the opioid prescription rates among injured workers in 2016–2018 to be less rather than more than older data. A limitation of the data is that we are unable to assess the payment source for the different opioid prescriptions to better address this question. Additionally, it is possible that the injured workers were receiving an opioid prescription for some reason other than the work-related injury. Given the temporal relationship with the injury, we do not consider this to have occurred frequently.

A further limitation of our data was that we are not able to assess opioid prescriptions among workers who had medical claims only, so we only were able to assess those workers with the more severe work-related injuries.

The use of a state-wide automated prescription system provides a more complete view of the prescription of opioids to injured workers. Providers responsible for back to work restrictions for safety sensitive workers need to be aware that individual workers may still being prescribed opioids even though there are no longer prescriptions being written within the workers’ compensation system. Further work to ensure that best practices are followed for opioid prescriptions needs to account that providers not associated with occupational medical programs maybe an ongoing source of opioid prescriptions.
